# The influence of core service quality associated with Korean women’s volleyball on national image and consumption engagement of Korean products: Perspective of Korean Wave in Thailand

**DOI:** 10.3389/fpsyg.2022.788257

**Published:** 2022-07-22

**Authors:** Jong-Hwan Jeon, Kevin K. Byon, Hyunseok Song, Sung-Bae Roger Park

**Affiliations:** ^1^Department of Sports Convergence, Kyungil University, Gyeongsan, South Korea; ^2^Department of Kinesiology, Indiana University Bloomington, Bloomington, IN, United States; ^3^Department of Sport Management, Wellness, and Physical Education, University of West Georgia, Carrollton, GA, United States; ^4^Department of Sport Industry, Hanyang University, Seoul, South Korea

**Keywords:** core service quality, national image, Korean Wave, stimulus-organism-response, behavioral intentions

## Abstract

Building on the stimulus-organism-response (SOR) framework, we proposed and tested a hypothesized model examining the effect of core service quality on national image and related behavioral intentions (i.e., intention to visit Korea and intention to purchase Korean products). Using 286 samples collected from Nakhon Ratchasima, Thailand, during the Korea-Thai Super Match, we conducted confirmatory factor analysis (CFA) and structural equation modeling (SEM) to examine the measurement model and the hypotheses, respectively. The results revealed that player attractiveness and emotional experience positively affected national image and behavioral intentions (i.e., intention to visit Korea and purchase Korean products). The findings stress that foreign spectators’ attitudes toward Korean women’s volleyball could translate into consumption behaviors (i.e., visits and Korean products) through the national image.

## Introduction

COVID-19 drastically impacted the sport industry in 2020. Nearly all sporting events were canceled or postponed, including the 2020 Tokyo Olympic Games. While most countries were under strict lockdowns during the pandemic, Korean sport leagues recommenced earlier and attracted considerable global attention from the sport media (Helin, 2020; Phuket News, 2020). With that attention, foreign sport fans followed Korean leagues *via* broadcasts by neighboring countries’ sport media. For example, K League, the Korean professional soccer league, broadcasted games live in several Asian countries (e.g., China, India, Singapore, Taiwan, Thailand) (League, 2020).

The public’s interest in Korean sport leagues has been enhanced by the Korean Wave (or “Hallyu” in Korean), referring to various phenomena in Korean popular culture that includes pop, drama, beauty, film and television, food, and fashion, which have gained immense popularity abroad (Ryoo, 2009; Son and Kijboonchoo, 2016). As globalization vastly influences politics, the economy, society, culture, lifestyle, and sport attendance, their popularity extends beyond their geographical boundaries to neighboring countries. For instance, Thai consumers who watched Korean dramas held a positive view of both the Korean national image (e.g., Korea’s economic development) and cognitive image (e.g., cultural and personal recognition). Consequently, their enhanced Korean image influenced their intention to purchase Korean cosmetics (Son and Kijboonchoo, 2016). In terms of sport industry, sport fans who positively influence the Korean Wave are likely to have a favorable attitude toward Korea overall and a higher positive perception of Korea’s national image than are non-fans of the Korean Wave (Jung, 2006).

The Korean Wave has significantly influenced the Korean economy, increased exports, and attracted tourists worldwide to Korea (Hogarth, 2013). The number of foreign tourists visiting Korea because of the Korean Wave has increased steadily, and the sales volume of products made in Korea, including cosmetics, fashion, and Korean cuisines, has skyrocketed. Recently, sport has been acknowledged as a significant factor in national competitiveness, creating a new economic value. It has become an essential element in cultural content and could become a primary agent supporting the Korean Wave syndrome in pop culture.

The Korean Wave of sport can be accomplished by developing icons and content whose core values align with Korean professional sports. The Korean Wave of sport phenomenon has become particularly popular in Southeast Asian countries like Vietnam and Thailand. For instance, Korea’s professional soccer league received TV ratings in Vietnam three times higher than that of Britain’s English Premier League (EPL) or Germany’s Bundesliga (Yoo et al., 2018). Although Korean sport attendance/viewing has increased among fans in neighboring countries, few studies have investigated why foreign fans engage with Korean sport leagues or the consequences associated with this engagement.

Sport marketing scholars have argued that core service quality in spectator sport (i.e., the game itself) is one of the main drivers of fan attendance at sport events (Byon et al., 2010, 2013; Jang et al., 2020). When sport fans attend these events, they are highly concerned with various core service qualities, such as the team’s performance, the excitement in its play, and the attractiveness of star players (Byon et al., 2010, 2013). When sport fans form their attitude toward attributes concerning core service quality related to a sport team, the attitude exerts the fans to develop internal organismic states associated with the attributes of the team, which will in turn influence the fans to engage in future consumption behaviors, such as reattending and recommending the sport game event they attended to others (Byon et al., 2013).

Sport fans’ behavioral processes can be explicated by the stimulus (S), organism (O), and response (R) model (SOR, Mehrabian and Russell, 1974), which posits that the environmental stimuli (e.g., core service quality related to a sport team) could induce sport fans’ internal organism (e.g., national image), which will create either approach or avoidance responses (e.g., visit Korea or Korean product purchase). Sport marketing scholars in diverse contexts have applied the SOR model to explain sport fan behaviors. For instance, Jang et al. (2020) examine the extent to which sportscape (i.e., physical service features associated with sport venues) exerts’ behavioral outcomes through emotion formation in the context of U.S. professional sport leagues. In the SOR framework, the authors found that there is a serial process in sport service quality (stimulus), positive emotion concerning the service quality (organism), and behavioral intention (R) in four U.S.-based major sport leagues (i.e., MLB, NBA, NFL, and NFL). Specifically, the authors found that service quality related to attending a sport league’s game (e.g., employees, wait time, venue aesthetics, scoreboard quality, layout accessibility) elicited sport fans’ positive emotions, which led them sequentially to reattend or recommend the sport event.

While previous studies have focused on core sport service quality’s effect on fans’ subsequent emotions and behavioral intentions to attend domestic sport events, international sport fans’ response to foreign sport leagues has been overlooked. Addressing this issue is particularly timely and relevant as sport fans consume sport products and services without any constraints, owing to innovation and technology advancement. Broadening marketing horizons for sport organizations has become a new trend in the sport industry. For instance, ESPN, a 24-h cable sports channel based in the United States, bought media rights to telecast several international events, including EPL, Formula 1, and Wimbledon, among others. This strategic decision for ESPN was aimed at increasing the cable’s subscription rates.

The National Football League (NFL) launched the NFL International Series in 2007, hosting several regular-season games in London and Mexico. As a result of the success, NFL and Tottenham Hotspur F.C. announced a 10-year deal to host at least two NFL games in Tottenham’s new stadium. These examples clearly show that effective utilization of core quality associated with sport entities (e.g., teams, leagues) could be a great marketing and tourism strategy for the sport teams (leagues) and the countries. However, only scant academic attention has been paid to test the effects of core quality on foreigners’ sport and tourism-related consumption. As such, we examined this issue in the current study.

The popularity of Korean sport leagues in the neighboring countries has surged due to the Korean Wave. The interest in Korean sport leagues on the part of sport fans in neighboring countries is an opportunity to broaden Korean sport leagues and related industries. From the tourism perspective, positive service experiences enhance the destination’s image and lead tourists to revisit (Jin et al., 2015; Tosun et al., 2015). Similar to the broad impact the consumption of pop culture has on the Korean national image and the intention of purchasing Korean products (e.g., the impact of K-drama consumption on Korean national image and the purchase of K-cosmetics; Son and Kijboonchoo, 2016), sport fans’ positive evaluation of the elements of their participation elicits a positive image of the sport team’s home country, and the positive emotions about the destination thus lead fans to visit the country or related products purchased produced there (Whang et al., 2016). We adopted the SOR framework to identify whether sport fans who support Korean sport leagues have a favorable view of the Korean national image and intend to visit Korea and purchase Korean products. Thus, the purpose of this study was to examine the effect of core service quality associated with the Korean women’s volleyball team on national image and related behavioral intentions (i.e., intention to visit Korea and intention to purchase Korean products) using samples collected from Nakhon Ratchasima, Thailand, during the Korea-Thai Super Match.

## Literature review

### Stimulus-organism-response

The sequential SOR chain has been adopted in sport consumer behavior studies to explain customers’ emotional and behavioral responses to sport activities (Jones et al., 2019; Jang et al., 2020; Kim et al., 2020; Teare et al., 2021). According to the framework, individuals respond positively (e.g., desire to explore, stay, engage) or negatively (e.g., no desire to revisit) to external stimuli (Jones et al., 2019; Kim et al., 2020). Sport service quality is also treated as an external stimulus, and sport service that fulfills sport spectators’ expectations of service quality elicit positive emotions about the service and subsequently evoke behavioral intentions (e.g., revisiting or recommending the event to others) (Jang et al., 2020; Teare et al., 2021).

For instance, Jang et al. (2020) examined the SOR framework concerning the relationship among the sportscape’s service quality (stimulus), positive emotion (organism), and behavioral intention (response) in U.S. major professional sport leagues (i.e., MLB, NFL, NBA, and NHL). The results showed that when sport event attendees experienced high service quality in the sportscape (i.e., friendly employees, reasonable wait times, aesthetic and clean venues, a quality stadium scoreboard, convenient access to facilities, and comfortable seats), these positive service quality experiences increased their positive emotions about the service, leading to their behavioral intention (i.e., revisit intention). Surveying spectators attending a cycling event, Teare et al. (2021) studied a track cycling event and found that watching sport events elicits spectators’ positive emotions (e.g., interest, alertness, excitement) and subsequent behavioral intention (i.e., attending the sport event).

Sport spectators’ subsequent emotions and behavioral intentions prompted by service quality are not limited to the sporting event itself. Sport fans also evaluate the overall image of an organization that provides a particular sport service (Alam and Noor, 2020). For instance, Alam and Noor (2020) found that service quality (stimulus) influences customers’ emotions associated with the service provider’s image (organism) and customers’ subsequent loyalty (response).

In this study, we applied the SOR framework to examine the relationships among service quality, sport spectators’ emotions, and behavioral intention concerning foreign sport league attendance behavior. As the study focused on sport consumers’ foreign sport league attendance, we investigated the service quality’s effect on country-related emotion (i.e., national image) and behavioral intention (i.e., revisit the sport league’s home country and purchase products made there).

### Core service quality in foreign sport league (stimulus) events

Core service quality’s influence on sport spectators’ behavior has been considered as a pivotal factor in sport consumer behavior research (Byon et al., 2010, 2013; Sarstedt et al., 2014). Service quality is an overarching term that includes multiple factors: team performance, star players, seating comfort, facility space, social event for fans, and regional bonds (Sarstedt et al., 2014). Sport marketing scholars have categorized service quality along two dimensions, core and peripheral (Byon et al., 2013). Core service quality is the service condition that fulfills fans’ expectations of the game itself (Byon et al., 2013). Conversely, peripheral service quality is the service condition related to the attributes of event support factors (i.e., game amenities, ticket service, and venue quality). Although both dimensions are considered as critical antecedents of spectators’ behavioral intentions (e.g., Byon et al., 2013), we are concerned only with core service quality in the current study. Concerning foreign sport league viewing behavior, sport fans’ experience is limited to the foreign teams’ away games in their own country or watching their matches *via* online media, when sport consumers experience only peripheral service. Thus, service quality in this study refers to core service quality. Following the factors identified previously and given the study’s context, three service quality dimensions were examined.

#### Dimension 1: Player attractiveness

Each player performs in all sport games, whether a spectator sport is a team (e.g., volleyball, basketball) or an individual sport (e.g., tennis, boxing). Scholars have considered that star players’ presence is a crucial team characteristic (Yoshida and James, 2010; Byon et al., 2013; Sarstedt et al., 2014). When more star players participate in a sport match, spectators’ satisfaction increases (Yoshida and James, 2010; Sarstedt et al., 2014). Grimshaw and Larson (2021) argued that a critical relationship between star players and sport match viewership. According to the literature, sport spectators recognize that superstars’ play increases a match’s entertainment value, which increases viewership (Grimshaw and Larson, 2021). In addition, Kim et al. (2020) also examined star player endorsements’ effect on fans’ sport brand passion (e.g., “I idealize this brand”) and loyalty (e.g., “I will be loyal to this brand for a long time”). Thus, player attractiveness is an essential factor when sports fans evaluate the core service quality of team performance.

#### Dimension 2: Emotional experience

Emotion is a core element that influences sport fans’ experience. In the sport engagement process, both positive and negative emotions affect perceived value associated with sporting events (e.g., watching a sport match) and subsequently influence sport spectators’ behaviors (e.g., purchase). Sport marketing scholars have suggested that various emotions (e.g., excitement, interest, entertainment) determine spectators’ engagement as supporters (Funk et al., 2002). For example, if a sport spectator evaluates a sport event experience as fun and exciting, s/he will tend to support and attend/view the sport event in the future (Jeong et al., 2020).

#### Dimension 3: Team performance

Team performance refers to the game’s outcome and is a pivotal determinant of core service quality (Byon et al., 2013). For example, a sport match’s quality concerning play and game management increases fans’ satisfaction and makes the game experience more enjoyable overall (Byon et al., 2010, 2013; Sarstedt et al., 2014). Not surprisingly, the quality of the game itself is one of the most critical attributes that affect a sport customer’s positive experience of the sport event. For instance, Byon et al. (2010) claimed that spectator sport fans demand to watch high-quality sport events and suggested that a sport team’s win/loss record, league standing, and high level of playing skills are among sport fans’ demands. Byon et al. (2013) also found that team performance-related elements (i.e., home team win/loss record, home team league standing, opposing team’s performance overall, quality of opposing team players, and of the opposing team overall) are the antecedents of the perceived value (e.g., “The game experience was fairly priced”). In turn, they directly affected sport consumers’ behavioral intentions (i.e., attendance and recommendation).

### National image in supporting foreign sport leagues (organism)

National image refers to country sport attendees’ mental image perceiving or recalling after attending a foreign sport event (Zeng et al., 2011). In the literature on mega-sport events (e.g., Olympics, FIFA World Cup) and sport tourism, the national image has been considered as a critical factor affecting sport fans’ experience (Zeng et al., 2011; Knott et al., 2013; Holtzhausen and Fullerton, 2015). For instance, Zeng et al. (2011) claimed that the 2008 Beijing Olympics heightened foreign sport fans’ interest and stimulated conversations about it in China. Holtzhausen and Fullerton (2015) compared the change in Americans’ perception of the 2010 FIFA World Cup host, South Africa, using a pre/post-quasi-experiment, applying 1,237 U.S. adult samples. The findings revealed that U.S. adults’ affection for South Africa improved after the 2010 FIFA World Cup. Knott et al. (2013) also examined the benefits of hosting the 2010 FIFA World Cup in terms of nation branding. The authors found that the majority of international sport tourists who spectated the 2010 FIFA World Cup in South Africa positively perceived the national brand of South Africa across several factors (e.g., beautiful scenery, national attractions, friendly people, a good climate for tourism and sport, a world-class tourism destination, well-respected political leaders, a safe place to visit).

### Behavioral intention (response)

Sport marketing scholars have focused on sport attendees’ behavioral intentions to measure their subsequent behaviors after experiencing a sport event (e.g., Byon et al., 2010, 2013). After attending a sport match, representative behavioral intentions on which previous scholars have focused include (1) intention to revisit the sport game and (2) word-of-mouth intention (e.g., recommending the sport game to other people) (Byon et al., 2010, 2013). In the international sport event context, behavioral intentions may be directed toward host country-related activities (Knott et al., 2013; Holtzhausen and Fullerton, 2015). Holtzhausen and Fullerton (2015) found that international sport fans who attended the 2010 FIFA World Cup had greater intention to revisit the country, recommend visiting the hosting country to others, and purchase the country’s products using a sample survey of U.S. adults. Similarly, Knott et al. (2013) found that most international visitors intended to revisit South Africa, encourage others to visit the country, and buy South African products more frequently.

### Core service quality’s effect on Korean national image and intention to visit Korea and purchase Korean products

According to the SOR framework, stimulus attributes affect the organism and response (Jang et al., 2020; Kim et al., 2020; Teare et al., 2021). In this study, we focused on the attributes of core service quality associated with the Korean women’s volleyball team by including player attractiveness, emotional experience, and team performance as the stimulus attributes (Byon et al., 2010, 2013; Sarstedt et al., 2014). In the sport marketing literature, the core service quality perceived at a sporting event affects sport fans’ evaluation of the service experienced (i.e., the perceived value of the sport match) and subsequent behavioral intentions (i.e., revisit and recommend) (Byon et al., 2010, 2013). Further, in the sport tourism and international sport management contexts, scholars have found that core service quality leads sport spectators to become attached to the destination and intend to patronize the location, which increases destination loyalty (Knott et al., 2013; Holtzhausen and Fullerton, 2015; Zhang et al., 2019).

Sport marketing and tourism scholars have suggested that core service quality directly influences consumer behavioral responses (e.g., Byon et al., 2010, 2013; Tzetzis et al., 2014; Jeong et al., 2019). For instance, Byon et al. (2010) argued that sport consumers develop attitudes toward attributes related to core service quality (e.g., the quality of the opposing team or the home team’s league standing). Using U.S. professional sport events spectators, the researchers found that core service quality positively influences reattending a sporting event. Byon et al. (2013) found that the core service attributes exerted sport consumption behaviors (e.g., traveling to attend events, word-of-mouth). Jeong et al. (2019) reported similar findings, demonstrating the direct impact of event quality (i.e., international marathon event) on behavioral intentions (i.e., traveling to the destination hosting the event and word-of-mouth of the event destination). Involving sport tourists traveling to an event destination, Tzetzis et al. (2014) supported the direct effect that contest quality affected sport tourists’ behavioral intentions of revisiting and recommending the event site and destination.

Based on the previous findings, we expected that forming perception for core service quality attributes of the Korean women’s volleyball team may have direct and indirect effects on behavioral intentions (i.e., visit Korea and purchase Korean products) through developing perception of national image Korea. Therefore, we have put forth the following hypotheses:

• *Hypothesis 1a: Player attractiveness is associated positively with Korea’s national image.*
• *Hypothesis 1b: Emotional experience is associated positively with Korea’s national image.*
• *Hypothesis 1c: Team performance is associated positively with Korea’s national image.*
• *Hypothesis 2a-1: Player attractiveness is associated positively with visiting Korea.*
• *Hypothesis 2a-2: Player attractiveness is associated positively with the intention to purchase Korean products.*
• *Hypothesis 2b-1: Emotional experience would be positively associated with the intention to visit Korea.*
• *Hypothesis 2b-2: Emotional experience would be positively associated with intention to purchase Korean products.*
• *Hypothesis 2c-1: Team performance is associated positively with the intention to visit Korea.*
• *Hypothesis 2c-2: Team performance is associated positively with the intention to purchase Korean products.*

### Korean national image’s effect on intention to visit Korea and purchase Korean products

Based on the SOR framework, the organism’s attributes serve as a moderator between the stimulus and response (Jang et al., 2020; Kim et al., 2020; Teare et al., 2021). Thus, the stimulus’s attributes affect both the response’s attributes directly and affect the response’s following attributes through the organism indirectly. In this study, the organism’s attribute is the national image, while the response’s attributes are the intention to visit and purchase. Scholars have found that the tourism destination’s image affects behavioral intention (i.e., recommendation, visit, and revisit) (Afshardoost and Eshaghi, 2020). Applying the findings of the previous literature and modifying it for this study, we hypothesized as follows:

• *Hypothesis 3a: Korea’s national image (Organism) is associated positively with the intention to visit Korea (Response).*
• *Hypothesis 3b: Korea’s national image (Organism) is associated positively with the intention to purchase Korean products (Response).*

**FIGURE 1:**
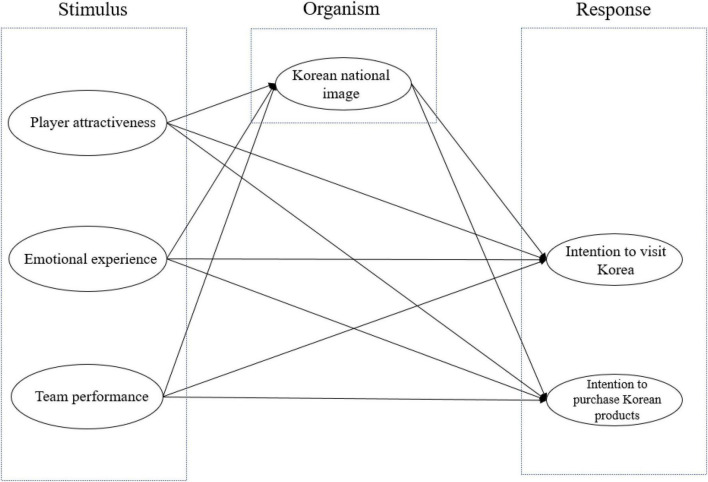
Research model.

The hypothesized model is exhibited in Figure 1.

## Materials and methods

### Measurement items

The questionnaire consisted of seven sections. Each of the three core service quality factors of Korean women’s volleyball (Funk et al., 2002; Byon et al., 2013; Kim and Kim, 2013) was measured using five items on a 7-point Likert-type scale anchored very unlikely (1) and very likely (7). Korean national image (Martin and Eroglu, 1993; Lee, 2015) was measured using five items on a 7-point Likert scale. By adapting Zeithaml et al.’s (1996) scale, we measured intention to visit Korea *via* six items. Intention to purchase Korean products was measured using four items on a 7-point Likert scale (Hagger and Chatzisarantis, 2005). Because we conducted the current study using a Thai sample, the questionnaire was made available in Thai. Since the questionnaire was drawn from measures initially developed in English, we used a back-translation method suggested by Brislin (1970). Specifically, the English version of the survey instrument was translated into Thai by a researcher who is a native speaker of Thai and fluent in English. Then, the Thai version was back-translated into English by a second bilingual speaker who was blind to the original form of the items. This process resulted in two versions of the survey in English. After that, both versions were compared and assessed whether the original meaning was retained during the translation and back-translation. The result of this comparison indicated that the two instruments were conceptually equivalent. Therefore, the use of the Thai version was deemed appropriate.

### Data collection

Data were collected at the Nakhon Ratchasima, Thailand, where the Korea-Thai Super Match was held on April 5, 2019. The capacity of the arena was 2,500. Efforts were made to approach fans in attendance by adopting systematic random sampling. Twenty trained assistants distributed surveys by intercepting every fifth person who entered the arena. If the intercepted fan declined to participate in the survey, the interviewer was trained to intercept the next person passing by until the interviewer identified an individual who agreed to participate in the survey. After the individual agreed, the study was presented in detail, and the fans were asked to complete the questionnaires on-site and return them when finished. The questionnaire took approximately 10–15 min to complete. A small gift branded with the match was given in exchange for their participation.

Out of 300 completed questionnaires, 14 were discarded due to the incompleteness of the responses. Two hundred eighty-six samples were used for the final analysis from 300 surveys. Ninety-six participants were male (33.6%), and 190 were female (66.4%). Looking at the distribution by age, 54.2% of respondents were in the age group of 20–29, and 54.2% of respondents were in the age group of 30–39, exceeding 70% of the total research subjects, with a large number of respondents from relatively young age groups. Concerning employment, students (37.1%), followed by business people (14.0%), employees (10.8%), and salespeople (10.8%). Regarding the average monthly income of respondents, around 70% were below Thailand THB 20,000–29,999. Table 1 summarizes the demographic profile of the respondents.

**TABLE 1:** Profile of respondents (*N* = 286).

Factors and variables	
**Gender**	
Male	33.6
Female	66.4
**Age**	
Under 19	16.1
20–29	54.2
20–29	17.5
40–49	8.4
Age 50 or older	3.8
**Occupation**	
Student	37.1
Company employee	1.8
Businessman/woman	14.0
Civil servant	7.3
Professional	6.3
Technician	3.1
Sales/service employee	10.8
Housewife	3.1
Other	
**Educational level**	
Less than high school	15.4
High school graduate	23.4
College graduate	57.7
Graduate school or above	3.5
**Annual income**	
Less than THB 10,000	3.8
THB 10,000–THB 19,999	18.2
THB 20,000–THB 29,999	48.3
THB 30,000–THB 39,999	16.8
THB 40,000–THB 49,999	9.1
THB 50,000 and above	3.8
**Marriage**	
Single	68.2
Married	30.1
Other	1.7
**Number of visits to Korea**	
0	81.8
1	13.3
2 or above	4.8

### Data analysis

Before conducting confirmatory factor analysis (CFA) to estimate the measurement model and structural equation modeling (SEM) for hypothesis testing, we examined assumption checks associated with CFA and SEM, including normality, multicollinearity, and outlier. Univariate normality was assessed *via* skewness and kurtosis, multicollinearity was identified through variance inflation factor (VIF), an outlier was examined *via* boxplot.

Upon verifying the assumptions, we tested the psychometric properties of the hypothesized model by following Anderson and Gerbing (1988) two-step modeling, in which a measurement model was estimated, followed by a structural model. The overall model fit was examined *via* adopting multiple fit indexes (i.e., χ*^2^*, CFI, TLI, RMSEA). To test construct validity, we examined convergent (*via* factor loading’s significance and magnitude) and discriminant validity (comparing AVEs and squared correlations, see Table 2). Following the verification of the measurement model, SEM was conducted to test the proposed model. The same fit indexes as used in the CFA were used to test the overall model fit of the structural model. Lastly, we examined the regression coefficients to test the hypotheses.

**TABLE 2:** Confirmatory factor analysis for validity and reliability test.

Factors and variables	Factor loading	AVE	CR	Cronbach’s α
**Player attractiveness**				
1. I like Korean women’s volleyball players	0.82	0.74	0.83	0.93
2. Korean women’s volleyball stars are my role model	0.93			
3. Korean women’s volleyball stars are attractive	0.94			
4. Korean women’s volleyball stars are good looking	0.56			
5. Korean women’s volleyball stars provide a friendly image to me	0.98			
**Emotional experience**				
1. Korean women’s professional volleyball is fun	0.71	0.55	0.83	0.83
2. I often watch Korean women’s professional volleyball games	0.79			
3. Watching Korean women’s professional volleyball game makes me happy	0.74			
4. Watching Korean women’s professional volleyball is always exciting	0.73			
**Team performance**				
1. I am satisfied with the Korean women’s professional volleyball league	0.93	0.56	0.78	0.77
2. I think the Korean women’s volleyball league is higher level than others	0.72			
3. I think Korean women’s professional volleyball teams play well	0.54			
**Korean national image**				
1. Korea is technologically developed country	0.97	0.81	0.85	0.94
2. Korea is an economically developed country	0.76			
3. Korea has a high standard of living	0.99			
4. Korea is a culturally advanced country	0.87			
**Intention to visit Korea**				
1. It would be exciting to visit Korea	0.92	0.86	0.96	0.96
2. It would be beneficial for me to visit Korea	0.91			
3. I prefer to visit Korea	0.88			
4. I will recommend friends and family to visit Korea	0.99			
**Intention to purchase Korean products**				
1. I like Korean products	0.90	0.73	0.91	0.91
2. I prefer to purchase Korean products	0.79			
3. I will recommend friends and family to purchase Korean products	0.84			
4. I will tell friends and family about the high quality of Korean products	0.88			

x^2^ = 600.74, df = 237, (x^2^/df = 2.535), GFI = 0.940, CFI = 0.947, NFI = 0.915, TLI = 0.938, RMSEA = 0.073.

## Results

### Assumption test

Regarding normality, the results revealed that skewness values ranged from −0.880 to 0.215, and the kurtosis values ranged from −1.190 to 0.976, both of which are within the acceptable ranges (Hair et al., 2010). These data showed the VIF ranged from 1.018 to 1.796, meeting the criterion of 3–5 suggested by Hair et al. (2010). Thus, there was no evidence of multicollinearity in the data set (Hair et al., 2010). Lastly, we conducted boxplots to identify outliers. Based on the results, outliers were not of concern. Therefore, proceeding to CFA for evaluating the psychometric properties of the measurement is deemed adequate.

### Measurement model

A CFA was conducted to examine the psychometric properties of the measurement model. The result of the CFA showed that the overall model fit was deemed adequate (*x*^2^ = 600.34, df = 237, *x*^2^/df = 2.533, CFI = 0.903, TLI = 0.938, RMSEA = 0.036). Convergent validity was examined *via* the significance and magnitude of factor loading. As a result of CFA, all factor loadings were statistically significant and above the suggested criterion of 0.50 (Hair et al., 2010), indicating that convergent validity of the measurement model was established.

The discriminant validity was tested based on the comparison of the AVE and squared correlation. As shown in Table 3, none of the squared correlations exceeded the AVE value, indicating discriminant validity was not a concern.

**TABLE 3:** The squared correlations and AVE of constructs.

	(1)	(2)	(3)	(4)	(5)	(6)
Player attractiveness (1)	0.74*a*					
Team performance (2)	0.26^b^	0.56				
Emotional experience (3)	0.67	0.33	0.55			
Korean national image (4)	0.61	0.27	0.64	0.81		
Intention to visit Korea (5)	0.72	0.32	0.63	0.64	0.86	
Intention to purchase Korean products (6)	0.67	0.23	0.67	0.64	0.70	0.73

^a^Average variance extracted (AVE) for each construct is displayed on the diagonal.

^b^Number below the diagonal are the squared correlation estimates between the two constructs.

### Hypotheses testing

The hypotheses were tested *via* SEM. The hypothesized model demonstrated a good overall fit (*x*^2^=487.32,*df* = 233,*x*^2^/df = 2.617, CFI = 0.944, TLI = 0.935, and RMSEA = 0.075).

**FIGURE 2:**
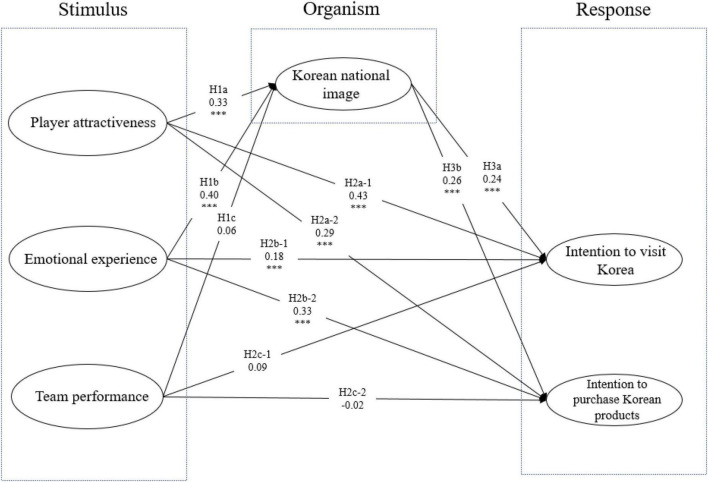
Result of structural equation model. Model Fit: x^2^ = 487.32, df = 233, x^2^/df = 2.617, TLI = 0.935, RMSEA = 0.075. ^***^p < 0.001.

The standardized path coefficient estimates of the hypothesized model appear in Table 4 and Figure 2.

**TABLE 4:** Results of structural equation modeling.

Path	Coefficient	*t*	*p*	Results
H1a: Player attractiveness - > Korean national image	0.326	4.87	0.000	Support
H1b: Emotional experience - > Korean national image	0.541	5.06	0.000	Support
H1c: Team performance - > Korean national image	0.059	1.11	0.265	Not support
H2a-1: Player attractiveness - > Intention to visit Korea	0.593	6.82	0.000	Support
H2a-2: Player attractiveness - > Intention to purchase Korean products	0.247	4.40	0.000	Support
H2b-1: Emotional experience - > Intention to visit Korea	0.338	2.47	0.013	Support
H2b-2: Emotional experience - > Intention to purchase Korean products	0.426	4.07	0.000	Support
H2c-1: Team performance - > Intention to visit Korea	0.128	1.96	0.050	Not support
H2c-2: Team performance - > Intention to purchase Korean products	−0.020	−0.41	0.683	Not support
H3a: Korean national image - > Intention to visit Korea	0.343	4.32	0.000	Support
H3b: Korean national image - > Intention to purchase Korean products	0.248	4.21	0.000	Support

The SEM results found that player attractiveness and emotional experience positively influenced Korea’s national image. Thus, H1a (β = 0.326, *t* = 4.866, *p* < 0.001) and H1b (β = 0.541, *t* = 5.056, *p* < 0.001) were supported. In terms of Hypothesis H1c, the path coefficient from the team performance factor to the Korean national image was not significant (β = 0.059, *t* = 1.114, *p* = 0.260), disconfirming H1c.

Testing the hypothesis about the intention to visit Korea revealed that player attractiveness and emotional experience positively influenced visiting the country. Thus, H2a-1 (β = 0.593, *t* = 6.822, *p* < 0.001) and H2b-1 (β = 0.338, *t* = 2.472, *p* < 0.05) were supported, but H2c-1 (β = 0.128, *t* = 1.959, *p* = 0.050), which is the impact of team performance on intention to visit Korea, was rejected as having no statistically significant impact.

The path coefficient from player attractiveness to intention to purchase Korean products (H2a-2: β = 0.274, *t* = 4.402, *p* < 0.001) and from emotional experience to intention to purchase Korean products (H2b-2: β = 0.426, *t* = 4.070, *p* < 0.001) were statistically significant, confirming H2a-2 and H2b-2. However, the path coefficient from team performance to intention to purchase Korean products (H2c-2: β = −0.020, *t* = −0.408, *p* = 0.683) was not significant, leading us to reject H2c-2.

Lastly, the path coefficient from Korean national image to intention to visit Korea (H3a: β = 0.343, *t* = 4.315, *p* < 0.001) and to intention to purchase Korean products (H3b: β = 0.248, *t* = 4.214, *p* < 0.001) were found to be statistically significant, confirming H3a and H3b.

## Discussion

This study examined the effect of core service quality on national image and related behavioral intentions such as the intention to visit Korea and purchase Korean products. The study was conducted in the context of Korean women’s volleyball performed in a foreign nation (i.e., Thailand). Although scholars have focused on the Korean Wave’s impact on the consumers of neighboring countries in various aspects (e.g., politics, economy, society, culture, lifestyle, sport), the impact on the sport industry has been superficially examined and compared with cultural effect. Specifically, while K-drama or K-pop were frequently referenced when explaining the Korean Wave’s impact (e.g., Ryoo, 2009; Son and Kijboonchoo, 2016), the impact of spectating K-sport leagues on the Korean national image and the intention to visit Korea was not examined. This study revealed that the impact of the Korean Wave omnidirectionally influences an increase Korean national image, and that the K-sport industry is also consistently influential.

Previous scholars recognized the impact of core service quality on sport consumers’ behavioral intentions when sport consumers spectate domestic sport games (Byon et al., 2010, 2013). Core service quality is constituted by factors determining sport game quality such as player attractiveness, positive emotion toward the experienced sport games, and sport team’s performance quality. Their findings propose that improved core service quality will increase sport fans’ behavioral intentions to attend sport games in the future (Byon et al., 2010, 2013). This study revealed the effect of core service quality through the national image of sport players or leagues toward sport consumers’ behavioral intention (e.g., visiting a foreign sport player’s home country and willingness to purchase the country’s products applying SOR framework in a foreign sport event consumption).

With the recently increased public interest in Korea, such as Korean Wave, the current study timely revealed that successful core service quality positively influences Korean national image and foreign sport consumers’ intentions to visit Korea and consume Korean products. In the current study, the dimensions of core service quality were classified explicitly in three dimensions: player attractiveness, emotional experience, and team performance. The hypothesized model addressed the impact of the three core service quality dimensions toward Korean national image and behavioral intentions (i.e., intention to visit Korea and willingness to purchase Korean products). The findings reveal that two dimensions of core service quality (i.e., player attractiveness and emotional experience) directly influenced behavioral intentions, and also the two dimensions indirectly influenced the behavioral intentions through Korean national image.

According to the indirect path of core service quality through national image toward behavioral intentions, the SOR framework was conceptualized. In the literature (Jang et al., 2020), while scholars have identified the role of the SOR framework in the relationship among peripheral service quality (i.e., servicescape), positive emotion, and intention to revisit sport events when spectators attend the U.S. professional leagues (i.e., MLB, NBA, NHL, and NFL), in the current study, SOR framework is applied to explained the impact of three core service dimensions (i.e., player attractiveness, emotional experience, and team performance) through national image toward behavioral intentions. When it comes to the foreign sport event, sport fans experience limited core service quality dimensions because the event is held in a foreign country, but at their home country. For instance, in the current study, a Korean women’s volleyball team played a match at Nakhon Ratchasima, Thailand. In this case, core service quality (i.e., player attractiveness, emotional experience, and team performance) could be focused on sport fans’ consumption.

The impact of player attractiveness on Korean national image, intention to visit Korea, and intention to purchase Korean products was proved in the results. Although volleyball is a team sport game, sport fans recognize an individual player in the team, and interest in famous players is an essential factor when sport fans follow spectating sport events (Funk et al., 2002). In the current study, the findings revealed that the impact of player attractiveness is not limited to attending the player’s sport event but is associated with the player’s national country and consumptions related to the country. Player attractiveness directly increases sport fans’ intention to visit the player’s country and purchase its products; it also indirectly increases them by boosting the country image. Emotional experience in Korean women’s professional volleyball also directly increases the intention to visit Korea and purchase Korean products. Foreign spectators who consume Korean women’s volleyball matches consider the sport exciting, and this emotion is positively associated with the Korean national image, intention to visit Korea, and intention to purchase Korean products. In terms of the impact of team performance, although the core service quality was hypothesized as an antecedent of Korean national image and following Korea-related consumption intention, the impact of team performance was not proved in the results. According to the results, foreign sport fans of Korean women’s volleyball are not concerned about the performance-based league level.

The findings are consistent with previous sport marketing scholars (Funk et al., 2002; Byon et al., 2010, 2013; Jang et al., 2020). Jang et al. (2020) are SOR framework of sportscape (S)—positive image (O)—behavioral intention to consume U.S. professional leagues (R) were extended to the association among core service quality (S)—Korean national image (O)—Korea-related behavioral intentions such as visiting Korea and purchasing Korean products (R). Following sport marketing scholars’ (Funk et al., 2002; Byon et al., 2010, 2013) claims that core service quality is associated with spectating sport consumption, the current study revealed the positive impact of player attractiveness and emotional experience toward intention to visit Korea and purchase Korean products. While previous studies examined the impact of core service quality on sport consumers’ intention in popular domestic sport leagues (e.g., Funk et al., 2002; Byon et al., 2010, 2013; Jang et al., 2020), the current study examined the impact of core service quality with the SOR framework in a less-popular sport league.

While sport marketing scholars applied spectators’ favorable attitude toward a player or a team to explain behavioral intentions such as game attendance, sport tourism scholars have focused on macroscopic behavioral aspects like national image and international travels in mega-sport events (Tzetzis et al., 2014; Jeong et al., 2019). The authors claimed that international spectators who attended the Olympic Games or FIFA World Cup developed positive attitudes toward revisiting the location (Tzetzis et al., 2014; Jeong et al., 2019). By echoing results of previous studies, we also found that foreign spectators’ behavioral intentions were actualized directly and indirectly through national image, players’ attractiveness (e.g., star players), and emotional experience.

Surprisingly, team performance, considered a significant core service quality in spectating sport events (Byon et al., 2010, 2013; Jeong et al., 2019), was not found to be significant in the current study. Possible reason for the insignificant result may be that spectating sport is based on competition-related play with professional skills, and sport fans expect aesthetics and dramatic situations generated by skilled performance (Funk et al., 2002). Thus, sport teams’ skill-based performance increases spectators’ perceived value, leading to their behavioral intention to reattend the game (Byon et al., 2013). Team performance was not significantly associated with national image or behavioral intentions.

Two findings were revealed in the results. First, team performance might not be associated with the national image or country-related behavioral intentions. Regarding the behavioral intentions to revisit a sport event, while the sport event is related to skill-based performance, sport players’ home-country product or visiting their country was unrelated to their skill performance. Second, sport fans might not be concerned with team performance in annual special events. For instance, sport fans’ motivations to watch an annual special match of a foreign sport league might focus on following specific sport stars or watching the particular game itself. To identify whether sport fans consider team performance in a specific situation such as an annual special event (e.g., NFL London match), researchers might need to examine the impact of team performance on national image and behavioral intentions to visit the players’ home country or purchase products of the country.

### Managerial implications

The findings in this study also provide practical implications. The findings provide that international sport fans’ interest in Korean sport leagues enhances Korea’s national image and, consequently, international fans travel to Korea and purchases Korean products. Global consumers’ interest in Korean culture, including but not limited to pop, drama, beauty, food, and sport. For instance, a sport journalist in Chinese media reported that Heung Min Son, an EPL player, is worth $1.8 billion to the South Korean economy (White, 2021). His fans purchased not only his EPL team jersey but also his national team jersey. Applying the current study’s findings, we suggest the importance of core service quality to increase Korean national image, sport consumers’ intention to visit Korea, and purchase intention toward Korean products with less popular sport leagues such as female volleyball. Specifically, increasing interest in individual sport players and focusing on the excitement of sport might enhance sport consumption related to the athlete’s home country. The current study revealed the impact of sport player attractiveness toward national image in country-related consumption behavior in a less-popular sport such as Korean women’s volleyball.

Although sport marketing using global star players is beneficial, the number of global stars is limited. However, the findings provide various sports and athletes might be applied to sport marketing, such as improving the national image and encouraging sport tourism. For instance, Yeon Kyoung Kim, a Korean professional volleyball player who played in neighboring countries (i.e., Japan, China, and Turkey), has one million fans following her on Instagram (Volleyball World, 2021). In major professional sports, sport tourism is already widespread. For instance, practitioners sell travel packages combining specific spectating sport events (e.g., MLB all-star game, Masters golf tournament) and travels. Although volleyball is not popular compared with the major professional sport leagues, the international sport fans are favorable to Kim, and sport marketing could focus on Kim’s attractiveness and her games to increase Korean national image and intentions to visit Korea. Concentrating on the marketing of two core services such as sport star marketing and promoting the excitement of Korean women’s volleyball games, sport tourism managers give shape to a plan of sport tourism. To maximize the effect of the Korean Wave, sport tourism managers and marketers can benchmark other Korean-celebrity-related marketing strategies. For instance, BTS, which is a K-pop band, also creates a massive economic impact. The team associated fans’ Korea travel with BTS’s music videos (Tamondong, 2020). Similarly, Korean volleyball player Kim might be focused on the star marketing associated with sport tour packages and Korean product advertisements. For instance, focusing on Kim’s story and Korean women’s volleyball games combined with Korean tourism attractions, foreign sport fans’ Korea tours, Korean women’s volleyball game attendance, and purchasing Korean products might be expected.

### Limitations and future directions

This study includes limitations that should be acknowledged. First and foremost, our results were derived from the data collected through a survey questionnaire. Although the hypothesized model was developed based on an established theory (i.e., SOR) and findings of previous studies related to core service quality and destination/national image and sport consumption, the relationships we examined are not causal but correlated. Therefore, a careful interpretation is needed as the findings are correlational. Although true experimental designs are the best way to establish the causation, a longitudinal survey design may be a preferred method to test the robustness of the findings, considering the nature of the international sport events held in the same place annually.

Second, we analyzed sport spectators who followed the Korean women’s professional volleyball in Thailand. Although women’s volleyball is a spectator sport, some distinct characteristics may be associated with fans following women’s volleyball games. For instance, volleyball is considered a less popular sport than other sports such as men’s basketball and soccer. Therefore, volleyball spectators’ characteristics might differ from those of significant sports. The cultural characteristics of Korea or Thailand might also influence these results. Although previous scholars (Saengchai and Jermsittiparsert, 2020) have suggested that the behavioral intentions of Thai consumers, as it relates to service quality, is described in general (e.g., consumers’ satisfaction and service provider’s quality positively influence loyalty), the potential uniqueness of Thai consumers’ characteristics might be overlooked. To enhance the generalizability of the findings in the current study, future studies should examine the hypothesized model using samples collected from different sports and cultures.

## Data availability statement

The raw data supporting the conclusions of this article will be made available by the authors, without undue reservation.

## Ethics statement

The studies involving human participants were reviewed and approved by Hanyang University Institutional Review Board. The patients/participants provided their written informed consent to participate in this study.

## Author contributions

JHJ contributed to conception, design of the study, data collection, and writing — original draft. KKB and HSS contributed to the review of literature, data analysis, editing of the original and revised manuscripts. SBRP supervised the entire process of the development and revision of the manuscript. All authors contributed to manuscript revision, read, and approved the submitted version.

## Conflict of interest

The authors declare that the research was conducted in the absence of any commercial or financial relationships that could be construed as a potential conflict of interest.

## Publisher’s note

All claims expressed in this article are solely those of the authors and do not necessarily represent those of their affiliated organizations, or those of the publisher, the editors and the reviewers. Any product that may be evaluated in this article, or claim that may be made by its manufacturer, is not guaranteed or endorsed by the publisher.

## References

[B1] Afshardoost M. Eshaghi M. S. (2020). Destination image and tourist behavioural intentions: a meta-analysis. *Tour. Manag.* 81:104154. 10.1016/j.tourman.2020.104154

[B2] Alam M. M. D. Noor N. A. M. (2020). The relationship between service quality, corporate image, and customer loyalty of Generation Y: an application of SOR paradigm in the context of superstores in Bangladesh. *Sage Open* 10 1–19. 10.1177/2158244020924405

[B3] Anderson J. C. Gerbing D. W. (1988). Structural equation modeling in practice: a review and recommended two-step approach. *Psycholog. Bull.* 103 411–423. 10.1037/0033-2909.103.3.411

[B4] Brislin R. W. (1970). Back-translation for cross-cultural research. *J. Cross-Cult. Psychol.* 1 185–216. 10.1177/135910457000100301

[B5] Byon K. K. Zhang J. J. Baker T. A. (2013). Impact of core and peripheral service quality on consumption behavior of professional team sport spectators as mediated by perceived value. *Eur. Sport Manag. Q.* 13 232–263. 10.1080/16184742.2013.767278

[B6] Byon K. K. Zhang J. J. Connaughton D. P. (2010). Dimensions of general market demand associated with professional team sports: Development of a scale. *Sport Manag. Rev.* 13 142–157. 10.1016/j.smr.2009.07.005

[B7] Funk D. C. Mahony D. F. Ridinger L. L. (2002). Characterizing consumer motivation as individual difference factors: augmenting the sports interest inventory (SII) to explain level of spectator support. *Sport Mark. Q.* 11 33–43.

[B8] Grimshaw S. D. Larson J. S. (2021). Effect of star power on NBA all-star game TV audience. *J. Sports Econ.* 22 139–163. 10.1177/1527002520959127

[B9] Hagger M. S. Chatzisarantis N. L. (2005). First-and higher-order models of attitudes, normative influence, and perceived behavioural control in the theory of planned behaviour. *Br. J. Soc. Psychol.* 44 513–535. 10.1348/014466604X16219 16368017

[B10] Hair J. F. Black W. C. Babin B. J. Anderson R. E. Tatham R. L. (2010). *Multivariate data analysis*, 7th ed. Upper Saddle River, NJ: Prentice-Hall.

[B11] Helin K. (2020). *On a bright note, Asian leagues are starting up again. NBC Sports.* Available online at: https://nba.nbcsports.com/2020/03/17/asian-leagues-are-starting-up-again/ (accessed date 17 March 2020).

[B12] Hogarth H. K. K. (2013). The Korean wave: an Asian reaction to Western-dominated globalization. *Perspect. Glob. Dev. Technol.* 12 135–151. 10.1163/15691497-12341247

[B13] Holtzhausen D. Fullerton J. (2015). The 2010 FIFA World Cup and South Africa: a study of longer-term effects and moderators of country reputation. *J. Mark. Comm.* 21 185–198. 10.1080/13527266.2012.740065

[B14] Jang W. Byon K. K. Yim B. H. (2020). Sportscape, emotion, and behavioral intention: a case of the big four US-based major sport leagues. *Eur. Sport Manag. Q.* 20 321–343. 10.1080/16184742.2019.1607521

[B15] Jeong Y. Kim E. Kim S. K. (2020). Understanding active sport tourist behaviors in small-scale sports events: stimulus-organism-response approach. *Sustainability* 12:8192. 10.3390/su12198192

[B16] Jeong Y. Kim E. Yu J. (2019). Determinants of behavioral intentions in the context of sport tourism with the aim of sustaining sporting destinations. *Sustainability* 11:3073. 10.3390/su11113073

[B17] Jin N. Lee S. Lee H. (2015). The effect of experience quality on perceived value, satisfaction, image and behavioral intention of water park patrons: new versus repeat visitors. *Internat. J. Tour. Res.* 17 82–95. 10.1002/jtr.1968

[B18] Jones C. W. Byon K. K. Huang H. (2019). Service quality, perceived value, and fan engagement: case of Shanghai Formula One racing. *Sport Market. Q.* 28 63–76. 10.32731/SMQ.282.062019.01

[B19] Jung H. S. (2006). The effects of consumer’s perception of the Korean wave (Hallyu) on Korean product purchase and country image in the Chinese market. *J. Cons. Stud.* 17 79–101.

[B20] Kim M. J. Lee C. K. Jung T. (2020). Exploring consumer behavior in virtual reality tourism using an extended stimulus-organism-response model. *J. Trav. Res.* 59 69–89. 10.1177/0047287518818915

[B21] Kim S. B. Kim D. G. (2013). Preliminary Controversial Issues on Hallyu, the Korean Wave in Sport. *J. Kor. Philosoph. Soc. Sport Dance* 21 45–58.

[B22] Knott B. Fyall A. Jones I. (2013). The nation-branding legacy of the 2010 FIFA World Cup for South Africa. *J. Hospit. Mark. Manag.* 22 569–595. 10.1080/19368623.2012.663155

[B23] League K. (2020). *International coverage of K League.* Available online at: https://kleague.com/contents/news/16968 (accessed date May 2020).

[B24] Lee C. D. (2015). The Study on the Usage Patterns of Chinese Consumers for Contents Related to the Korean Wave. *J. Korea Cult. Ind.* 15 101–110. 10.5392/JKCA.2015.15.05.515

[B25] Martin I. M. Eroglu S. (1993). Measuring a multi-dimensional construct: country image. *J. Bus. Res.* 28 191–210. 10.1016/0148-2963(93)90047-S

[B26] Mehrabian A. Russell J. A. (1974). *An approach to environmental psychology.* Cambridge, MA: MIT Press.

[B27] Phuket News (2020). *Professional sport returns in South Korea. Phuket News.* Available online at: https://www.thephuketnews.com/professional-sport-returns-in-south-korea-75787.php (accessed date April 2020).

[B28] Ryoo W. (2009). Globalization, or the logic of cultural hybridization: the case of the Korean wave. *Asian J. Comm.* 19 137–151. 10.1080/01292980902826427

[B29] Saengchai S. Jermsittiparsert K. (2020). Determining the loyalty of customers with moderating role of service quality: a study on Thailand. *Internat. J. Innov. Creat. Change* 11 188–203.

[B30] Sarstedt M. Ringle C. M. Raithel S. Gudergan S. P. (2014). In pursuit of understanding what drives fan satisfaction. *J. Leis. Res.* 46 419–447. 10.1080/00222216.2014.11950335

[B31] Son S. Kijboonchoo T. (2016). The impact of Korean wave on the purchase intention of Korean cosmetics of Thai people in Bangkok and Chonburi, Thailand. *PSAKU Internat. J. Interdiscipl. Res.* 5 76–83. 10.12778/235108618X15452373185705

[B32] Tamondong H. (2020). *10 must-visit places in Seoul for every BTS fan. Cosmopolitan.* Available online at: https://www.cosmo.ph/entertainment/must-visit-places-in-seoul-for-bts-fans-a4575-20200201-lfrm (accessed date February 2020).

[B33] Teare G. Potwarka L. R. Snelgrove R. Drewery D. (2021). Inspiring participation in a new sport opportunity: exploring the role of event experience and spectator characteristics. *Event Manag.* 25 227–244. 10.3727/152599519X15506259856291 30089248

[B34] Tosun C. Dedeoğlu B. B. Fyall A. (2015). Destination service quality, affective image and revisit intention: the moderating role of past experience. *J. Dest. Mark. Manag.* 4 222–234. 10.1016/j.jdmm.2015.08.002

[B35] Tzetzis G. Alexandris K. Kapsampeli S. (2014). Predicting visitors’ satisfaction and behavioral intentions from service quality in the context of a small-scale outdoor sport event. *Internat. J. Event Fest. Manag.* 5 4–21. 10.1108/IJEFM-04-2013-0006

[B36] Volleyball World (2021). *One million fans follow Kim on Instagram.* Available onlilne at: https://en.volleyballworld.com/volleyball/competitions/olympics-2020/news/one-million-fans-follow-kim-on-instagram (accessed date August 2021).

[B37] Whang H. Yong S. Ko E. (2016). Pop culture, destination images, and visit intentions: theory and research on travel motivations of Chinese and Russian tourists. *J. Bus. Res.* 69 631–641. 10.1016/j.jbusres.2015.06.020

[B38] White J. (2021). *Squid game? BTS? Song Heung-min should be atop the ‘Korean Wave’. South China Morning Post.* Available online at: https://www.scmp.com/sport/football/article/3151876/squid-game-bts-son-heung-min-should-be-atop-korean-wave (accessed date October 2021)

[B39] Yoo S. K. Lim C. H. Chang J. (2018). Media Portrayal of Foreign Coaches in Korea and Vietnam. *Korean J. Comm. Stud.* 26 27–45. 10.23875/kca.26.4.2

[B40] Yoshida M. James J. D. (2010). Customer satisfaction with game and service experiences: antecedents and consequences. *J. Sport Manag.* 24 338–361. 10.1123/jsm.24.3.338

[B41] Zeithaml V. A. Berry L. L. Parasuraman A. (1996). The behavioral consequences of service quality. *J. Mark.* 60 31–46. 10.1177/002224299606000203

[B42] Zeng G. Go F. Kolmer C. (2011). The impact of international TV media coverage of the Beijing Olympics 2008 on China’s media image formation: a media content analysis perspective. *Internat. J. Sports Market. Sponsorship* 12 39–56. 10.1108/IJSMS-12-04-2011-B004

[B43] Zhang J. Byon K. K. Williams A. S. Huang H. (2019). Effects of the event and its destination image on sport tourists’ attachment and loyalty to a destination: the cases of the Chinese and US Formula One Grand Prix. *Asia Pacific J. Tour. Res.* 24 1169–1185. 10.1080/10941665.2019.1667837

